# Disease-mediated bottom-up regulation: An emergent virus affects a keystone prey, and alters the dynamics of trophic webs

**DOI:** 10.1038/srep36072

**Published:** 2016-10-31

**Authors:** Pedro Monterroso, Germán Garrote, Ana Serronha, Emídio Santos, Miguel Delibes-Mateos, Joana Abrantes, Ramón Perez de Ayala, Fernando Silvestre, João Carvalho, Inês Vasco, Ana M. Lopes, Elisa Maio, Maria J. Magalhães, L. Scott Mills, Pedro J. Esteves, Miguel Ángel Simón, Paulo C. Alves

**Affiliations:** 1 CIBIO/InBio, Centro de Investigação em Biodiversidade e Recursos Genéticos, Universidade do Porto, Campus Agrário de Vairão, 4485-661, Vairão, Portugal; 2 Agencia de Medio Ambiente y Agua de Andalucía, C/Johan G. Gutenberg. 1, 41092, Seville, Spain; 3 Instituto da Conservação da Natureza e das Florestas, Av. da República, 161050-191, Lisbon, Portugal; 4 IESA-CSIC, Instituto de Estudios Sociales Avanzados, Plz Campo Santo de los Mártires. 7, 14004 Córdoba, Spain; 5 WWF/España, Gran Vía de San Francisco 8-D, 28005, Madrid, Spain; 6 Fundación CBD-Habitat, C/ Gustavo Fernández Balbuena 2, Entreplanta, Oficina A, 28002, Madrid, Spain; 7 ANPC, Associação Nacional Proprietários Rurais, Gestão Cinegética e Biodiversidade, R. Mestre Lima de Freitas, 1-5°, 1549-012, Lisbon, Portugal; 8 Departamento de Biologia, Faculdade de Ciências, Universidade do Porto, R. Campo Alegre s/n, 4169-007, Porto, Portugal; 9 Wildlife Biology Program, University of Montana, 32 Campus Drive, Missoula, MT 59812, USA; 10 Consejería de Medio Ambiente de la Junta de Andalucía, C/ Doctor Eduardo García-Triviño López 15, 23071 Jaén, Spain

## Abstract

Emergent diseases may alter the structure and functioning of ecosystems by creating new biotic interactions and modifying existing ones, producing cascading processes along trophic webs. Recently, a new variant of the rabbit haemorrhagic disease virus (RHDV2 or RHDVb) arguably caused widespread declines in a keystone prey in Mediterranean ecosystems - the European rabbit (*Oryctolagus cuniculus*). We quantitatively assess the impact of RHDV2 on natural rabbit populations and in two endangered apex predator populations: the Iberian lynx (*Lynx pardinus*) and the Spanish Imperial eagle (*Aquila adalberti*). We found 60–70% declines in rabbit populations, followed by decreases of 65.7% in Iberian lynx and 45.5% in Spanish Imperial eagle fecundities. A revision of the web of trophic interactions among rabbits and their dependent predators suggests that RHDV2 acts as a keystone species, and may steer Mediterranean ecosystems to management-dependent alternative states, dominated by simplified mesopredator communities. This model system stresses the importance of diseases as functional players in the dynamics of trophic webs.

Substantial changes in ecosystems can be caused by species whose impact on its community far exceeds its biomass, often described as keystone species^1^. Similarly, emergent diseases may dramatically affect ecosystems, by creating new biotic interactions and modifying existing ones^2^. Despite the scarce evidence supporting host-species’ extinctions driven by infectious diseases^3^, the decline of a keystone host produces catastrophic cascading effects on whole ecosystems through altered predator-prey dynamics, mutualistic and other community interactions^3^,^4^,^5^. Infectious diseases may influence the dynamics of species interactions along trophic webs, potentially causing regime shifts in the ecosystem, especially if the etiological agent is new to the host species^2^,^4^,^6^. Likewise, if new diseases become enzootic they may cause declining phases in host populations, altering predator-prey dynamics, and thus causing major disturbances in trophic webs.

Mediterranean ecosystems within the Iberian Peninsula (IP) are important hotspots of biodiversity^7^, harboring several endemic species, including highly threatened apex predators like the Iberian lynx (*Lynx pardinus*) and the Spanish Imperial eagle (*Aquila adalberti*). Both endemic predators are dependent on the European rabbit (*Oryctolagus cuniculus*)^8^ a multifunctional keystone species in Iberian Mediterranean ecosystems^9^. Rabbit populations in Iberia have sharply declined since the 1950s, mainly as a consequence of viral diseases and habitat loss^10^, with consequential dramatic range contraction of rabbit-dependent predators^11^. One of the main limiting factors of Iberian rabbit populations is the rabbit hemorrhagic disease (RHD), which is caused by a calicivirus (RHDV), and showed initial mortalities of 55–75% at the end of the 1980s^12^. RHD became enzootic and mortality rates decreased, although continuing to play an important role in the dynamic of European rabbit populations^13^. Until 2011, all RHDV strains circulating in natural European rabbit populations belonged to the same genogroup (G1). However, a new variant of RHDV (RHDV2 or RHDVb) reached Spain in 2011^14^, and Portugal in 2012^15^. Currently, it has been the only etiological agent detected in Iberian rabbits, suggesting that it might have completely replaced G1 strains^16^,^17^. Although the rapid spread of this new variant has been associated with further rabbit population declines^18^,^19^, its impact on Mediterranean trophic webs has not been assessed.

By affecting a keystone species, the emergence of RHDV2 may have severely affected key ecosystem processes; for example, affecting trophic relations of rabbit-dependent predators^18^. This situation assumes particular relevance in the current context of Iberian lynx recovery and conservation. The Iberian lynx is the most threatened cat species in the world, and has become extinct in several areas of its historical range^8^, in some of which it is currently being reintroduced^20^. The Spanish Imperial eagle Portuguese breeding population went extinct in the 1980s^21^. Following its range recovery since the 1970s, it recolonized Portugal in 2003 and slowly established two main population nuclei^22^,^23^. In this study our goal is to quantitatively assess the impact of RHDV2 on rabbit populations using data from two areas representative of the carnivore and raptor guilds in the Mediterranean ecosystems of Iberia: Sierra de Andújar and Guadiana Valley regions, respectively; and to evaluate the functional impact of rabbit decline on endangered endemic apex predators. Based on our own data and on a literature review on the interspecific interactions in Mediterranean trophic webs, we discuss how RHDV2 has the potential to change the Mediterranean ecosystem by limiting the population size of its main prey species.

## Results

From January 2012 to October 2015, we analysed 85 samples (38.1%; n = 223) from rabbits carcasses found in the field, and 138 (61.9%; n = 223) from apparently healthy animals (from hunting activity and roadkills). All RHDV-positive samples were identified as RHDV2. We obtained an overall virus prevalence of 83.5% in animals found dead in the field and 10.9% in animals derived from hunting or roadkill (Table 1).

We obtained information about when carcasses were found in 61 (85.9%) of positive cases. Most of these of RHDV2-positive animals (n = 58; 85.9%) were found during the European rabbit’s breeding season (November to March). Furthermore, 54.9% (n = 28) of all animals found dead in the field for which we could accurately determine the age were <6 months old.

**Figure 1:**
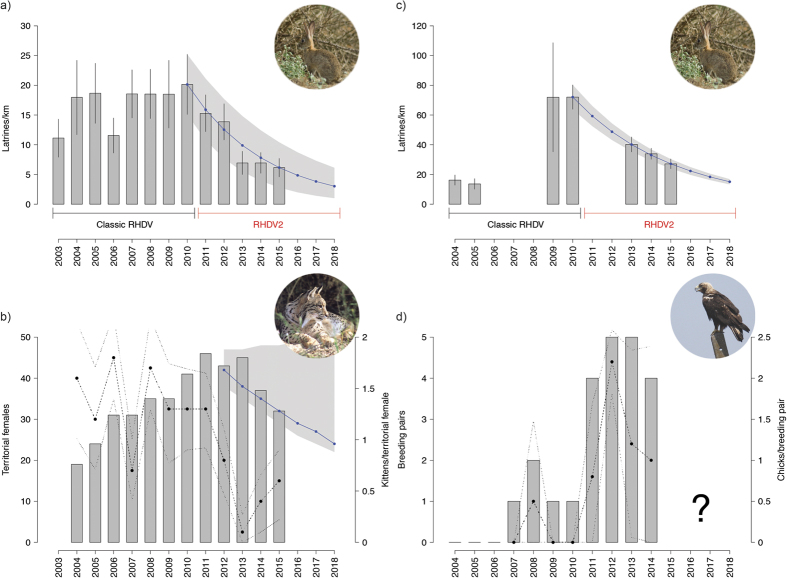
European rabbit (*Oryctolagus cuniculus*) abundance estimated by latrines counts (bar plot) at: (**a**) Sierra de Andújar, and (**c**) Guadiana Valley. Predators’ breeding populations estimates and fecundity: (**b**) Iberian lynx (*Lynx pardinus*) territorial females (bar plot), and correspondent mean fecundity, given by the number kittens produced per territorial female (dot plot) in Sierra de Andújar. (**d**) Spanish Imperial eagle (*Aquila adalberti*) breeding pairs (bar plot), and correspondent mean fecundity, given by the number chicks produced per breeding pair (dot plot) in Guadiana Valley. Results are presented as means ± 95% CI. Blue lines (and shaded area) indicate the projections (and 95% CI) of population growth for each species based on the models developed for the period after the arrival of RHDV2. Pictures courtesy of CIBIO/InBIO (European rabbit), Alfonso Moreno (Iberian lynx) and Carlos Pacheco (Spanish Imperial eagle).

The European rabbit population at Sierra de Andújar (SA) slightly increased from 2003 to 2010, the pre-RHDV2 period, with 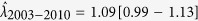 (Fig. 1). Following the arrival of RHDV2 in the area the population decreased 69.4% in five years, from 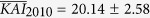 latrines/km to 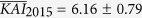 latrines/km, at a mean annual growth rate of 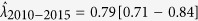.

The relative abundance of the European rabbit population in Guadiana Valley (GV) increased more than fourfold from 2004 to 2010, prior to the arrival of RHDV2; =16.25 ± 1.75 latrines/km and 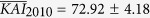 latrines/km, with an average annual growth rate of 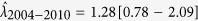. Following the arrival of RHDV2, rabbits’ population trajectory revealed a consistent decline from 2010 to 2015, with 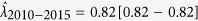. The relative abundance value decreased 44.2% from 2010 to 2013, and continued to drop continuously in the following years, with the abundance in 2015 (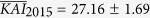 latrines/km) being 37.7% of what it was in 2010.

Our exponential growth models developed for RHDV2 period suggest that, under the current rate of decline, the relative abundance of European rabbits is expected to decrease in three years time to predicted values of 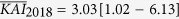 latrines/km at SA, and 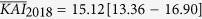 at GV (Fig. 1), only 21% and 15% of the relative abundance estimates prior to the arrival of this new etiological agent, respectively.

**Figure 2:**
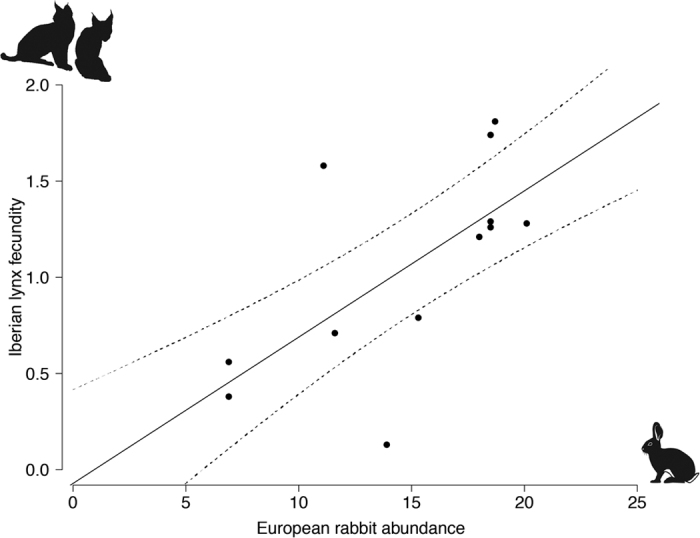
Relationship between Iberian lynx (*Lynx pardinus*) fecundity, given by the number of kittens born per territorial female per year, and European rabbit (*Oryctolagus cuniculus*) abundance, expressed as the number of latrines per kilometer, in Sierra de Andújar.

From 2004 to 2011, the female breeding population of Iberian lynx in SA was gradually increasing at a rate of 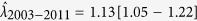, and presented a mean fecundity of 1.36 ± 0.12 kittens/year/territorial female (Fig. 1). However, the territorial female population started to decline with a one-year lag after the arrival of RHDV2 (Fig. 1). There was a 30.4% reduction in the number of territorial females between 2011 and 2015, resultant of a decreasing growth rate of 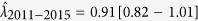. In spite of the high variability derived from the limited dataset, our projections suggest that, if conditions remain unchanged, the number of territorial females is expected to decrease to 24[22–50] individuals, nearly half (58%) of the breeding population size in 2011. The Iberian lynx fecundity decreased 89.6% from 2011 to 2013, to 0.13 ± 0.07 kittens/year/territorial female following the arrival of RHDV2. Overall, the mean Iberian lynx fecundity significantly declined by 65.7% from the pre to post-RHDV2 arrival period (1.36 ± 0.12 *vs* 0.47 ± 0.14; *W* = 31, *p* = 0.008). This drastic reduction prompted emergency management actions to increase lynx reproduction (see methods section), namely restocking with European rabbits and activation of supplementary feeding stations. The management actions had a significant effect in recovering lynx fecundity in the years of 2014 and 2015, as females occupying intervened territories had significantly higher fecundity than those in unmanaged ones (0.80 ± 0.20 *vs* 0.21 ± 0.11; *W* = 402, *p* = 0.005). As a result, the mean lynx fecundity slightly recovered after 2013 to 0.56 ± 0.17 kittens/year/territorial female. Still, the mean reproductive output of a territorial female in 2015 was 56.1% lower than in 2011. The model that included the relative abundance of European rabbit as explanatory covariate was significantly better than the null model (Robust Wald Test: w = 38.561, p < 0.001), and indicated that the relative abundance of European rabbits explained 48.5% of the observed variance in yearly lynx fecundity (*β* = 0.076 ± 0.01, *t* = 6.21, *p* < 0.001; Fig. 2).

A breeding population of the Spanish Imperial eagle started to establish naturally in 2007 in GV, and gradually increased during the following years at a rate of 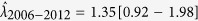 (Fig. 1). Fecundity accompanied this trend, and increased up to a mean value of 2.2 ± 0.36 chicks/pair in a total of five breeding pairs in 2012, the year when RHDV2 arrived in the area. Thereafter the number of breeding pairs declined to four by 2014, resulting in a potentially declining state. Given the reduced dataset for the RHDV2 period, our modelling estimates lacked precision (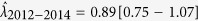), precluding reliable projections of population growth. Regardless, we found a positive, but non-significant, relation between the Spanish Imperial eagle breeding population size and rabbit abundance at time t-1 (*ρ* = 0.79, *S* = 4.19, *p* = 0.11). The rabbit decline had an immediate impact, decreasing the average eagle fecundity by 45.5% from 2012 to 2013, to 1.2 ± 0.31 chicks/pair; and by 16.7% from 2013 to 2014, to 1.0 ± 0.28.

## Discussion

All RHDV-positive rabbits were infected with RHDV2, which is in agreement with other studies in the Iberian Peninsula^16^,^17^. The majority of the rabbits found dead in the field were RHDV2-positive while for hunted and roadkilled rabbits these were a minor fraction (10.9%). Because we did not know the age distribution in the affected rabbit populations, we could not assess the relative importance of RHDV2-related mortality in each age class. Nevertheless, over half of all RHDV2-positive carcasses found in the field were <6 months old rabbits confirming the susceptibility of young animals to the disease^14^,^15^. In spite of presenting very different European rabbit relative abundances prior to the arrival of RHDV2, the rabbit populations at both areas exhibited highly coherent declining rates coincident with the emergence of RHDV2, suggesting that the RHDV2-induced declining trends in natural European rabbit populations might be irrespective of their initial abundance. Our results are parallel to those reported in other Iberian populations^18^,^19^, and present a similar scenario to what was observed following the impacts of the first RHDV outbreaks^10^,^12^, with the difference that young rabbits consist of a target population class for RHDV2, compromising the recruitment of new individuals for natural populations.

Our results suggest that the emergence of RHDV2 had severe indirect negative impacts in two endangered endemic apex predators, the Iberian lynx and Spanish Imperial eagle. The near-extinction status of the Iberian lynx prompted the implementation of conservation efforts, which led to a 140% increase of its range and 171% in population size between 2002 and 2010^20^. Alongside, the recovery of the Spanish Imperial eagle that has been occurring at least since the 1970s^22^, was disrupted in the period of 1993–1999, following the arrival of RHDV^22^. Our results suggest that the Iberian lynx and Spanish Imperial eagle responded to the arrival of RHDV2 with decreased fecundity, which was previously observed after the first outbreak of RHDV in the 1990s^24^. An alternative hypothesis could be that the reduction of predator fecundity results from a density-dependent regulatory effect^25^. However, the fact that emergency management actions (i.e. European rabbit restocking operations and activation of supplementary feeding stations) have prompted a significant increase in lynx fecundity in SA in the years of 2014 and 2015 strongly suggests that the observed changes in the reproductive output is in fact the result of the availability of feeding resources. Our model also supports that rabbit abundance remains as the key factor related to Iberian lynx fecundity. This relation is further supported by the fact that, despite the RHDV2-induced declines, some lynx reintroduction areas in Andalusia (Spain) that retained relatively high rabbit densities enabled lynx to maintain reasonably unchanged fecundity levels (M.A. Simón, unpublished data).

If RHDV2 continues to induce the decline of European rabbit populations, a reduction in these apex predators’ population size and range is plausible. When RHDV first arrived in the 1980s, the estimated total Iberian lynx population size was ~1100 individuals, with 350 breeding females^26^. In 2010, just before the RHDV2 outbreak, it consisted of 252 individuals, with 68 breeding females^20^. This suggests that the impact of RHDV2 on lynx populations might be even higher than that of the classical RHDV. It should also be highlighted that our study area (SA) encompasses the main relict population of this species^20^,^27^. Regarding the Spanish Imperial eagle, the recovery in population size and range expansion in recent years allowed the establishment of renewed peripheral nuclei, such as the one at GV^23^. These nuclei are particularly fragile, and indeed our results suggest a breeding success decline in these re-established population after the rabbit crash caused by RHDV2. While these impacts may not threaten the short-time survival of this species, they may lead to the local extinction of peripheral nuclei and revert the recent positive demographic and distribution trend of the eagle.

**Figure 3:**
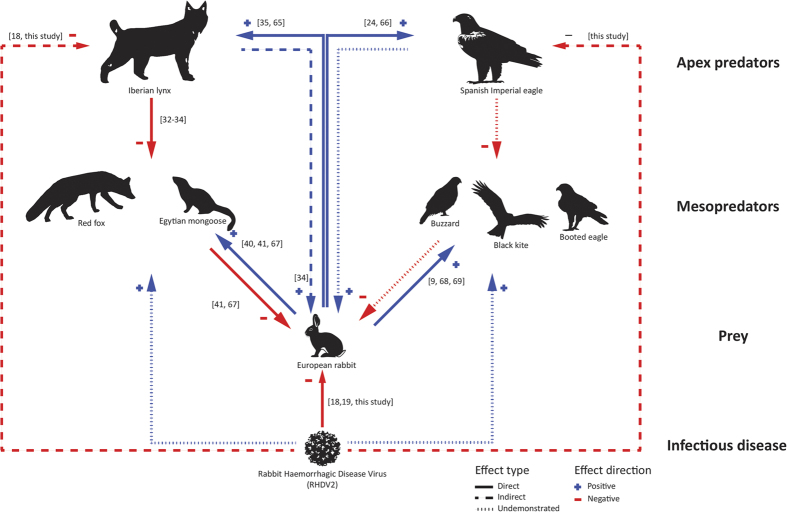
Diagram displaying published and potential interspecific relations between different levels in a vertebrate Mediterranean trophic web based on own data and published literature: Apex predators, mesopredators, prey and infectious disease. Numbers in square brackets correspond to references.

The recovery and re-establishment of apex predator populations contribute not only to their conservation, but also to the restoration of disrupted ecosystem processes^28^,^29^, particularly trophic interactions. The main interspecific relations in Mediterranean mammal and avian trophic webs, and how they could have been affected by RHDV2, are depicted in Fig. 3. Established top-predator populations influence mesopredators directly (through intraguild predation, IGP, and interspecific killing, IK) or indirectly^28^,^30^, excluding them from the most beneficial habitats or suppressing their populations^31^, which can have beneficial effects on prey populations^28^. A wide range of apex and mesopredators in the Iberian Peninsula are active European rabbit consumers^9^. The Iberian lynx enforces top-down control over Iberian mesocarnivores, namely red foxes (*Vulpes vulpes*), Egyptian mongooses (*Herpestes ichneumon*) and common genets (*Genetta genetta*)^32^,^33^ (Fig. 3), with indirect positive effects for rabbit populations^34^. Given its strong dependence on European rabbits, Iberian lynxes are unable to shift prey-base^8^,^35^ and therefore a negative numeric response is expected following rabbit declines, with consequent release of mesocarnivores from top-down control^28^,^31^. The more generalist trophic spectra of most Iberian mesocarnivore species^9^,^36^ enable these species to thrive under low availability of the European rabbit.

Bottom-up forces also play a critical role in shaping the structure of trophic webs^37^. While in high latitude ecosystems this bottom-up control is driven by natural demographic fluctuations in prey abundance^38^,^39^, in the Iberian Mediterranean system it appears to be essentially controlled by disease outbreaks; for example, the first outbreak of RHDV strongly contributed to the contraction of Iberian lynx populations^11^,^27^, resulting in the downgrading of Mediterranean ecosystems to a state dominated by simplified mammal communities of generalist species such as foxes and mongooses^36^. A twisted effect of this simplification of the ecological system is that foxes are able to maintain rabbits under a predator-pit effect^40^,^41^, further dampening the ecosystem and preventing is restoration^36^. Avian raptor guilds also exhibit highly structured communities, where intraguild aggression and predation often occurs^42^,^43^. Such interactions are driven by the limitation in the availability of a shared prey and its frequency is size-based^42^,^43^. In the Iberian Peninsula, it is likely that the Spanish Imperial eagle enforces top-down regulation of smaller raptors that consume rabbits regularly (i.e. the common buzzard *Buteo buteo*, the booted eagle *Hiearaaetus pennatus* and the black kite *Milvus migrans;* Fig. 3), as IK has the potential to structure whole raptor assemblages^42^. Therefore, there is a potential for RHDV2-driven disruptive effects in avian guilds, mirroring that of the mammalian community.

Similar examples of ecosystem-wide impacts of infectious diseases are found worldwide in terrestrial trophic webs. In the 1890s’ a rinderpest pandemic reduced wildebeest and buffalo populations in the Serengeti (Tanzania), and led to a reduction in the overall grazing pressure with consequent increase of larger fires, which suppressed the establishment of tree species. When rinderpest was eradicated in the 1960s, this whole process was reversed: the intense grazing reduced fire frequency, which allowed woodlands to regenerate, with consequent increase in large predators due to an improvement of vegetation cover, and hence, hunting success^44^. Likewise, anthrax (*Bacillus anthracis*) was responsible for over 50% of all herbivore deaths in the Etosha National Park (Namibia) in 1966–74^45^ causing fundamental changes in vegetation structure, and providing large amounts of carcasses that led to changes in the structure of the herbivore-carnivore-scavenger food webs^6^,^46^. More recently, in North America, the sylvatic plague (*Yersinia pestis*) steered black-footed ferret (*Mustela nigripes*) populations to the extinction in the wild directly via infection, and indirectly, via depletion of the prairie dog (*Cynomys* spp.)^47^, its staple prey. However, the bottom-up effects driven by this disease were contained in the trophic webs as they only reached the mesopredator level. These examples illustrate how diseases can precipitate regime shifts in ecological systems, especially if they affect keystone species or guilds^6^.

Our results suggest that, in Mediterranean ecosystems, RHDV2 currently falls into this category, because it affects directly host populations, and indirectly several other species all the way up to the apex predator level, hence playing a key role in shaping ecosystem processes and structure. By controlling the population dynamics of European rabbits, RHDV2 becomes an ecosystem engineer itself and therefore should be included as a functional player in the dynamics of terrestrial Mediterranean trophic webs. The recent emergence of this new variant constrains our data to relatively small time series, which is reflected in a reasonable degree of uncertainty associated with the inference provided by our population growth models. Regardless, the recent history of European rabbit populations suggests that the current extent of RHDV2 is likely to cause important declines in the populations of the highly threatened Iberian lynx and the Spanish Imperial eagle, potentially imperilling the conservation efforts and reversing their recent positive population trends.

## Materials and Methods

### Study areas

This study was performed in two strategic areas for the conservation of the Spanish Imperial eagle and Iberian lynx populations: the Guadiana Valley region (GV; Portugal; −7.8220°W, 37.7244°N to −7.6490°W, 37.6217°N), and Sierra de Andújar (SA; Spain; −4.25801°W, 38.30136°N to −3.88460°W, 38.12413°N). GV encompasses some of the highest European rabbit densities in Portugal, and currently fosters an Iberian lynx reintroduction program. This area comprises a surface of 17,341 ha in SE Portugal, is partially included in the Guadiana Valley Natural Park, and harbours the SW outmost population of the Spanish Imperial eagle. SA embraces the area occupied by the largest remaining Iberian lynx population and is where RDHV2 was first detected in an Iberian wild rabbit population^48^. It comprises 18,125 ha. This area is partially included in the Cardeña y Montoro and Sierra de Andújar Natural parks (SE Spain). Although the Iberian lynx population has expanded since, we only considered its fraction encompassed in the area occupied since the beginning of the study (i.e. 2003), for accurate comparative purposes. Both study areas are included in the Mediterranean basin biodiversity hotspot^7^ and have populations of the Spanish Imperial eagle, although breeding only started in 2007 at GV. The raptor community present at GV is representative of the raptor guild in the Mediterranean region of the IP, including species such as the golden eagle (*Aquila chrysaetos*), Bonelli’s eagle (*Hiearaaetus fasciatus*), booted eagle (*Hieraatus pennatus*), red kite (*Milvus milvus*) and common buzzard (*Buteo buteo*). Likewise, the carnivore community at SA is representative of the mammalian carnivore guild in the Mediterranean region of the IP, including species such as the European wildcat (*Felis silvestris*), red fox (*Vulpes vulpes*), stone marten (*Martes foina*), Eurasian badger (*Meles meles*), common genet (*Genetta genetta*) and Egyptian mongoose (*Herpestes ichneumon*).

### Virus isolation and sequencing

From January 2012 to October 2015, liver samples were obtained from rabbit carcasses found in the field, and from rabbits shot by hunters or roadkilled. The RNA extraction and the screening for RHDV strains by PCR amplifications were conducted as described in Lopes *et al*.^17^. For PCR-positive samples, the gene encoding the capsid protein VP60 was fully amplified by PCR and sequenced as in Dalton *et al*.^49^. Sequences were aligned in BioEdit version 7.0.9.0^50^ and compared with publicly available sequences including representatives of genogroups 1–6, RHDV2, non-pathogenic and weakly pathogenic strains.

### European rabbit population monitoring

The European rabbit populations in SA and GV were monitored using latrine counts along predefined transects^51^. Latrine counts are an indirect method frequently used to estimate European rabbit relative abundance across large areas^52^,^53^. It is the only feasible method for employment across large spatial scales, and is significantly and monotonically related to rabbit population density^54^. SA was sampled yearly from 2003 to 2015 with constant effort: 187.39 km sampled, corresponding to an effort of 0.77 km/km^2^. GV was sampled in 2004, 2005, 2009, 2010, 2013, 2014 and 2015 with a mean yearly distance sampled of 56.8 ± 9.76 km, corresponding to an average effort of 0.33 ± 0.06 km/km^2^. The number of active latrines was counted along predefined transects, established along trails, and stratified by habitat type. The European rabbit relative abundance was calculated using a kilometric abundance index (KAI) of latrines^53^. The overall index of annual abundance in the study area was estimated by averaging the KAI over all transects performed that year, which were sampled once a year at the end of European rabbits’ breeding season (June-July), corresponding to the period of highest density^55^, before the beginning of the hunting season.

### Spanish Imperial eagle: breeding population and fecundity

The apex predator monitored in the GV study area was the Spanish Imperial eagle. The Portuguese Institute for the Conservation of Nature and Forests (ICNF) provided the official data on this species breeding population monitoring and fecundity for the period of 2003–2014^23^. Briefly, starting in January, the area was surveyed weekly to locate nests when they were being built. After detection, monitoring activity consisted of recording breeding pairs’ behaviour (building nest or incubating), as well as the number of chicks during their development. Monitoring stopped when chicks left the nests. Nest monitoring was performed at a distance ≥500 m from the nest to avoid disturbance interference^24^. Fecundity was defined as the number of chicks per breeding pair.

### Iberian lynx: breeding population and fecundity

The apex predator monitored in the SA study area was the Iberian lynx, which was surveyed using camera-trapping methods as described in Gil-Sánchez *et al*.^56^ and Garrote *et al*.^57^. Surveys were carried out yearly in the dry season - May/June to November/December - to maximize cub detection. Camera-trapping stations were placed at the same point in the grid each year, at an approximated density of one station/km^2^ and a mean inter-camera distance of 0.87 ± 0.02 km, and were kept active for a period of 2 to 5 months, depending on efficiency and redundancy of captures. Cameras were baited with lynx urine or with live animals. Each camera trap was visited once a week for troubleshooting and care for live bait. Lynx were individually identified from the photographs using their coat patterns^56^,^57^. Given the intensive monitoring since 2002 and the small population size of Iberian lynx, all adult population is individually identified at SA^20^,^56^. Therefore, we were able to estimate the absolute number of territorial females in our study area from camera-trapping records. Breeding dens were not visited after parturition to assess litter size, to avoid disturbing breeding females. Instead, fecundity was assessed yearly by camera trapping through counting the number of kittens that accompanied each adult female after the breeding season. Therefore, our measure of fecundity regards to the number of kittens per territorial female detected during the period of post-weaning dependence. We identified a lynx as territorial if the individual had breeding status, determined through cub detection, and/or if the camera-trapping results supported a non-overlapping intrasexually minimum convex polygon 100 (MCP100)^56^,^58^.

No animals were killed or captured for the purpose of this study. All monitoring protocols were purely observational, i.e. did not involve animal capture and manipulation, and were carried out in accordance with the EU directive 2010/63/EU of 22 September 2010 on the protection of animals used for scientific purposes. All protocols were approved by the relevant institution: the Institute for the Conservation of Nature and Forests (ICNF, Portugal) or the Andalusia Ministry of Environment (Consejería de Medio Ambiente de la Junta de Andalucía, Spain).

### Data analysis

The study period was divided in two stages: pre-RHDV2, from 2003 to 2010; and RHDV2, from 2010 to 2015. We considered 2011 as the turning year in SA (Spain) and 2012 in GV (Portugal), because these were the years when the virus was first reported in each country^14^,^15^. The demographic trajectories of the European rabbit, Iberian lynx and Spanish Imperial eagle were evaluated by estimating the mean population growth for each species in the pre-RHDV2 and RHDV2 stages. We fitted models of stochastic exponential growth rate using the diffusion approximation approach, which allows for missing data in the time series and accounts for process variance arising from stochastic fluctuations^59^,^60^. Values of *λ* = *1* suggest a stationary population, whereas *λ* < 1 and *λ* > 1 values indicate declining and increasing population trends, respectively^60^.

Given that the Spanish Imperial eagle breeding population only established in GV in 2007, the reduced amount of data available precluded modelling the relationship between rabbit abundance and eagles’ breeding population and fecundity in this study area. Instead, we evaluated if there was an association between the number of breeding pairs and the European rabbit relative abundance using a Spearman rank correlation test.

We evaluated changes in Iberian lynx fecundity associated with both RHDV2 presence and European rabbit abundance. The mean fecundity per territorial female in the pre and RHDV2 arrival periods was compared using the Mann–Whitney U-test^61^. We fitted a robust linear regression model^62^ to evaluate the relationship between mean Iberian lynx fecundity at year *t* and the mean rabbit abundance (at year *t-1*). The robust linear regression is not sensitive to outliers in the response variable as it uses MM-estimation^62^. This regression was implemented using the package *robustbase* v0.92-5^63^ in R software^64^. Data are presented as mean ± SE and ranges (in square brackets) consist of 95% confidence intervals, unless explicitly stated otherwise.

## Additional Information

**How to cite this article**: Monterroso, P. *et al*. Disease-mediated bottom-up regulation: An emergent virus affects a keystone prey, and alters the dynamics of trophic webs. *Sci. Rep.* **6**, 36072; doi: 10.1038/srep36072 (2016).

**Publisher’s note:** Springer Nature remains neutral with regard to jurisdictional claims in published maps and institutional affiliations.

## Figures and Tables

**Table 1:** European rabbit samples collected during the period of 2012–2015, and respective results from RHDV screening.

Origin	Year	Negative	Positive	Total	Proportion positive
Found dead	2012	0	0	0	—
	2013	2	16	18	88.9%
	2014	7	34	41	82.9%
	2015	5	21	26	80.1%
	Total	14	71	85	83.5%
Hunted / Roadkill	2012	10	0	10	—
	2013	20	2	22	9.1%
	2014	57	10	67	14.9%
	2015	36	3	39	7.7%
	Total	123	15	138	10.9%
